# Activated PI3K delta syndrome 1 mutations cause neutrophilia in zebrafish larvae

**DOI:** 10.1242/dmm.049841

**Published:** 2023-03-13

**Authors:** Stone Elworthy, Holly A. Rutherford, Tomasz K. Prajsnar, Noémie M. Hamilton, Katja Vogt, Stephen A. Renshaw, Alison M. Condliffe

**Affiliations:** ^1^ Department of Infection, Immunity and Cardiovascular Disease, University of Sheffield, Sheffield S10 2TN, UK; ^2^ Department of Evolutionary Immunology, Institute of Zoology and Biomedical Research, Jagiellonian University, Gronostajowa 9, 30-387 Krakow, Poland

**Keywords:** APDS, Zebrafish, PI3K delta, Homology-directed gene editing, CRISPR, Cas12a, Cpf1, Neutrophil, Neutrophilia

## Abstract

People with activated PI3 kinase delta syndrome 1 (APDS1) suffer from immune deficiency and severe bronchiectasis. APDS1 is caused by dominant activating mutations of the *PIK3CD* gene that encodes the PI3 kinase delta (PI3Kδ) catalytic subunit. Despite the importance of innate immunity defects in bronchiectasis, there has been limited investigation of neutrophils or macrophages in APDS1 patients or mouse models. Zebrafish embryos provide an ideal system to study neutrophils and macrophages. We used CRISPR-Cas9 and CRISPR-Cpf1, with oligonucleotide-directed homologous repair, to engineer zebrafish equivalents of the two most prevalent human APDS1 disease mutations. These zebrafish *pik3cd* alleles dominantly caused excessive neutrophilic inflammation in a tail-fin injury model. They also resulted in total body neutrophilia in the absence of any inflammatory stimulus but normal numbers of macrophages. Exposure of zebrafish to the PI3Kδ inhibitor CAL-101 reversed the total body neutrophilia. There was no apparent defect in neutrophil maturation or migration, and tail-fin regeneration was unimpaired. Overall, the finding is of enhanced granulopoeisis, in the absence of notable phenotypic change in neutrophils and macrophages.

## Teaser

**Summary:** This study uses zebrafish embryos and homology-directed gene editing to investigate the effect of activated PI3 kinase delta syndrome 1 mutations on innate immune cells, observing neutrophilia without defects in neutrophil differentiation or migration.

## INTRODUCTION

Activated PI3 kinase delta syndrome 1 (APDS1) is a complex syndrome of immune system defects (Angulo et al., 2013; Lucas et al., 2014, 2016; Michalovich and Nejentsev, 2018; Singh et al., 2020; Brodsky and Lucas, 2021). Previous investigations of human APDS1 patients and mouse models have revealed T-cell and B-cell defects, but it is currently unclear whether there are also neutrophil or macrophage defects (Brodsky and Lucas, 2021).

APDS1 is caused by dominant gain-of-function mutations in the *PIK3CD* gene that encodes the PI3 kinase delta (PI3Kδ) catalytic subunit (p110δ) (Jou et al., 2006; Angulo et al., 2013; Lucas et al., 2014). PI3Kδ and its paralogues (PI3Kα and PI3Kβ) comprise the Class IA PI3 kinases (Burke and Williams, 2015). These are all heterodimers of their respective catalytic domain proteins with a common regulatory domain protein (p85 encoded by *PIK3R1*). In response to transmembrane receptor signalling, they phosphorylate the membrane phospholipid Ptdlns(4,5)P_2_ to generate the second messenger Ptdlns(3,4,5)P_3_. PtdIns(3,4,5)P_3_ activates a downstream kinase cascade, including AKT, that modulates mTOR and FOXO1, controlling differentiation, metabolism and proliferation (Burke and Williams, 2015; Lucas et al., 2016; Nunes-Santos et al., 2019). The Ptdlns(3,4,5)P_3_ phosphatases, PTEN and SHIP1 (also known as INPP5D), provide a reverse activity crucial for overall function of the signalling pathway (Nishio et al., 2007; Choorapoikayil et al., 2014; Browning et al., 2015).

PI3Kδ is predominantly expressed in haemopoietic stem and progenitor cells (HSPCs) and leukocytes (Burke and Williams, 2015). In human HSPCs, lymphocytes and neutrophils, *PIK3CD* expression is more abundant than expression of the *PIK3CA* and *PIK3CB* paralogues (encoding PI3Kα and PI3Kβ) (Xie et al., 2021). Human monocytes and myelocytes strongly express *PIK3CD*, along with *PIK3CB* (Xie et al., 2021).

PI3Kδ has diverse roles in modulating the differentiation and activity of different leukocytes (Okkenhaug et al., 2002; Lucas et al., 2016; Nunes-Santos et al., 2019). During emergency granulopoiesis in response to bacterial infection, PI3Kδ activity induces HSPCs to proliferate and differentiate to provide extra neutrophils (Kwak et al., 2015; Mistry et al., 2019). To provide adaptive immunity, PI3Kδ activity in B cells and T cells needs to be fine-tuned for various stages of differentiation. This level of PI3Kδ activity is set by signalling from antigen receptors, co-stimulatory receptors, cytokine receptors and growth factor receptors (Lucas et al., 2016; Nunes-Santos et al., 2019). Neutrophil migration and production of reactive oxygen species is controlled by PI3Kγ (PI3 kinase class 1B) (Hirsch et al., 2000; Li et al., 2000; Sasaki et al., 2000; Hannigan et al., 2002; Yoo et al., 2010). A role for PI3Kδ in these processes has also been implicated but is less clearly established (Sadhu et al., 2003; Condliffe et al., 2005; Liu et al., 2007; Sapey et al., 2014).

Studies of APDS1 patients identified B-cell and T-cell defects resulting from dysregulated excessive PI3Kδ activity (Angulo et al., 2013; Lucas et al., 2014). The most prevalent causative APDS1 mutation is E1021K in the p110δ catalytic domain and the second-most prevalent is E525K in the helical domain. E1021K is predicted to enhance membrane association, whereas E525K is predicted to perturb, regulatory interactions with the p85 regulatory partner (Dornan et al., 2017; Michalovich and Nejentsev, 2018). These alleles lower the PI3K activation threshold and mirror oncogenic mutations at equivalent positions of PI3Kα. The PI3Kδ inhibitor Leniolisib offers a promising treatment option (Rao et al., 2017). Mouse models of the E1021K mutation have further elucidated the mechanisms underlying the B-cell and T-cell defects (Avery et al., 2018; Stark et al., 2018; Wray-Dutra et al., 2018) and, more generally, the role of PI3Kδ in lymphocytes. In addition to disrupting adaptive immunity, aberrant PI3Kδ signalling in such mice expands a subpopulation of B cells that increase immediate susceptibility to *Streptococcus pneumoniae* infection (Stark et al., 2018).

Severe inflammatory airway damage (termed bronchiectasis) is a prominent APDS1 symptom and possibly suggestive of additional defects of the innate immune system (Condliffe and Chandra, 2018). However, there has been limited investigation of neutrophils and macrophages in APDS1 patients or mouse models (Chiriaco et al., 2017; Stark et al., 2018).

Zebrafish embryos provide an excellent model system for investigating neutrophil and macrophage development and function. The consequence of hyperactive PI3K signalling on myelopoiesis has previously been studied using zebrafish lacking Pten (Choorapoikayil et al., 2014). Lack of Pten induces an expansion of HSPC, and, although definitive myeloid and lymphoid lineages enter early stages of development, differentiation fails to complete (Choorapoikayil et al., 2014). Neutrophils from the primitive wave of myelopoiesis fully differentiate and are more numerous, whereas neutrophils from the definitive wave differentiate to the point of expressing *mpx* but not to having granules that can be stained with Sudan Black dye (Choorapoikayil et al., 2014).

The zebrafish *PIK3CD* orthologue, *pik3cd*, has been characterised as a *runx1*-dependent, early marker of HSPCs (Bonkhofer et al., 2019). Expression of *pik3cd* is observed in nascent HSPCs as they differentiate from the dorsal aorta and in the posterior blood island (Bonkhofer et al., 2019). Single-cell mRNA sequencing studies of zebrafish larval stages and adults also show extensive expression of *pik3cd* in neutrophils, macrophages and HSPCs, along with extensive expression of *pik3cg* and *pik3cb* encoding zebrafish Pi3kγ and Pi3kβ (Athanasiadis et al., 2017; Tang et al., 2017; Farnsworth et al., 2020).

Here, we used homology-directed gene editing of zebrafish *pik3cd* to generate the first zebrafish hyperactivating PI3Kδ APDS1 models and investigate possible primary defects in neutrophils or macrophages at a developmental stage prior to the development of the zebrafish adaptive immune system.

## RESULTS

### Homology-directed CRISPR-Cas9 knock-in of a zebrafish model for *PIK3CD^E525K^*

The zebrafish genome sequence assembly (GRCz11) has one *pik3cd* gene encoding an orthologue of the human *PIK3CD*. DNA sequence database searching confirmed that the closest *pik3cd* paralogues are genes encoding PI3Kβ, PI3Kγ and PI3Kα catalytic subunits (TBLASTN search of GRCz11 with human PIK3CD protein sequence). This indicated that zebrafish *pik3cd* could provide a suitable model for investigating mechanisms of human APDS1.

Zebrafish *pik3cd* has extensive ammino acid sequence homology to human *PIK3CD*, with conservation of the residues mutated in the two most prevalent APDS1 disease-causing mutations E1021K and E525K (Howe et al., 2013). We sought to mimic these by using gene editing to generate zebrafish *pik3cd* alleles with the equivalent E1017K and E525K mutations.

**Fig. 1.**
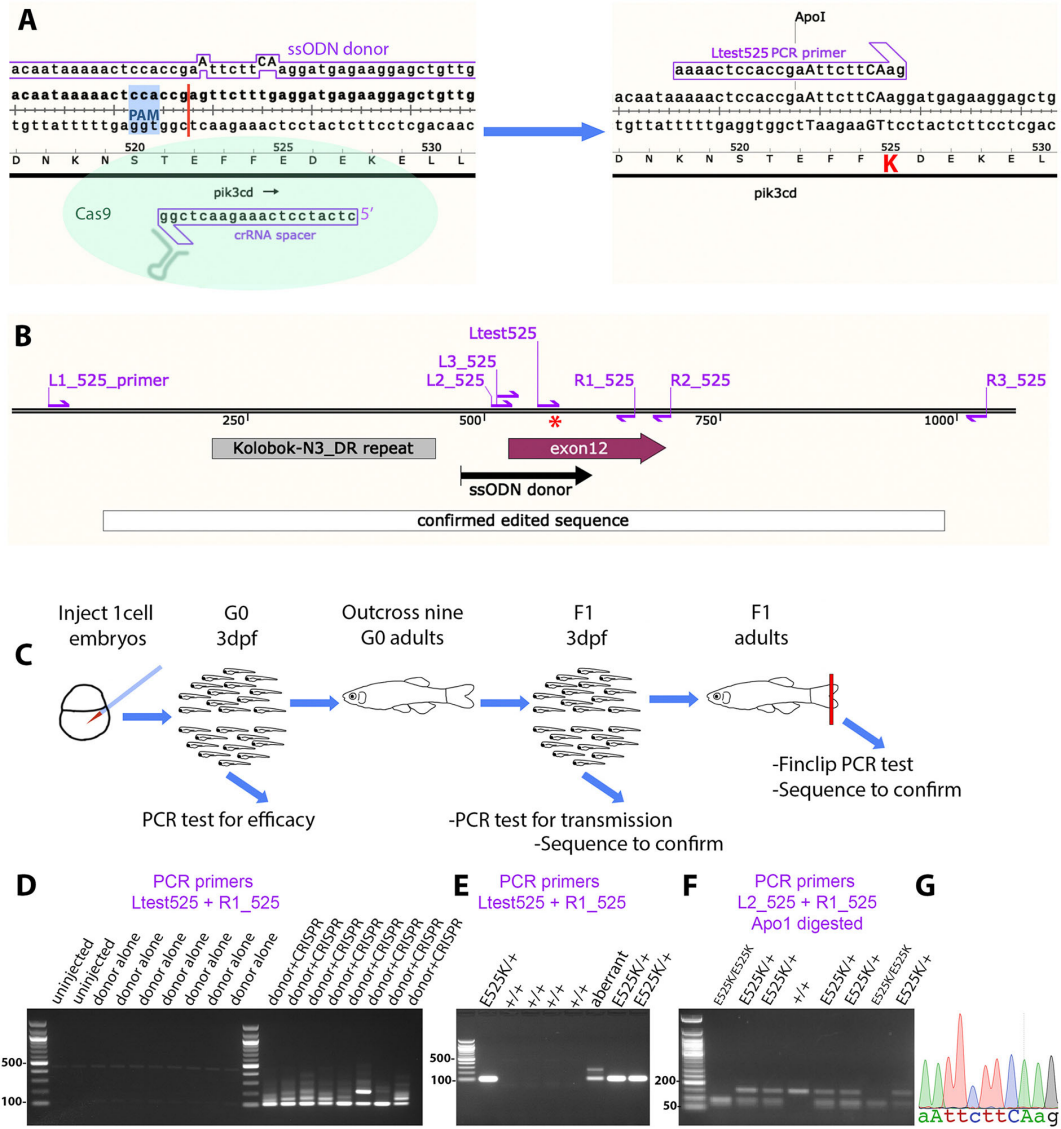
**Homology-directed CRISPR-Cas9 knock-in of a *pik3cd^E525K^* allele.** (A) Diagram illustrating the knock-in process. The CRISPR-Cas9 cleavage and knock-in is illustrated on the left and the resulting *pik3cd^E525K^* allele on the right. On the left, the single-strand oligonucleotide (ssODN) donor sequence and crRNA spacer sequence are positioned alongside the *pik3cd* DNA sequence and translated amino acid sequence, with the CRISPR-Cas9 protospacer adjacent motif (PAM) highlighted in blue and the cleavage site shown as a red line. On the right, mutated DNA bases are shown in upper case, the E525K coding change is highlighted in red, and the introduced ApoI cleavage site is indicated. The Ltest525 PCR primer is specific for the knock-in allele. (B) Map of the zebrafish *pik3cd^E525K^* locus. PCR primers are shown as magenta arrows; the mutation site is marked with a red asterisk; and positions are shown for the ssODN donor used for knock-in, the region confirmed by sequencing after knock-in, *pik3cd* exon 12 and a DNA repeat incompatible with PCR primer location. (C) Diagram illustrating the workflow for establishing the line with the *pik3cd^E525K^* allele. (D) Agarose electrophoresis gel with knock-in-specific test PCRs from 3 days post fertilisation (dpf) G0 embryos. All the embryos previously injected with CRISPR-Cas9 and ssODN donor produced the expected 103 bp amplicon and also lower electrophoretic mobility products. PCRs from uninjected embryos, or embryos injected with CRISPR-Cas9 alone, did not amplify. DNA size marker is a 100 bp ladder. (E) Agarose electrophoresis gel with knock-in-specific test PCRs from 3 dpf F1 embryos from an outcross of a G0 adult that had been injected with CRISPR-Cas9 and ssODN donor when an embryo. PCRs from embryos with the *pik3cd^E525K^* allele produced a 103 bp amplicon. A lower-mobility band indicates a putative aberrant knock-in event. DNA size marker is 100 bp ladder. (F) Agarose electrophoresis gel with Apo1-digested PCR amplicons from F2 embryos from a *pik3cd^E525K/+^* in-cross. The *pik3cd^E52K5^* allele introduces an Apo1 site, allowing *pik3cd^E525K/E525K^*, *pik3cd^E525K/+^* and *pik3cd^+/+^* genotypes to be distinguished. DNA size marker is 50 bp ladder. (G) Sanger sequencing trace from a PCR amplicon across the knock-in locus of a *pik3cd^E525K/E525K^* F2 adult. The mutated DNA bases are shown in upper case as in A.

Single-strand oligonucleotide (ssODN)-directed gene editing with CRISPR-Cas9 has recently become established as a method for knocking in point mutations in the zebrafish genome (Prykhozhij and Berman, 2018; Prykhozhij et al., 2018). Editing efficiency relies on having an efficient CRISPR cleavage site within 10 bp of the intended mutation (Prykhozhij and Berman, 2018). Searching with CRISPR-Cas9 design software revealed that the zebrafish *pik3cd* E1017 site (equivalent to the human *PIK3CD* E1021) lacks nearby potential CRISPR-Cas9 cleavage sites. We were, however, able to design a guide RNA for a potential cleavage site 8 bp from the *pik3cd* E525 site (Fig. 1A,B). These practicalities caused us to first direct our efforts towards the *pik3cd* E525 site, despite its lower clinical significance.

To knock in the *pik3cd^E525K^* mutation, we followed recommendations (Prykhozhij et al., 2018), designing an asymmetric, 140mer, template-strand, ssODN donor, with two phosphothioates at each end. The ssODN donor sequence had two further silent point mutations in addition to the E525K-causing point mutation (Fig. 1A). These were designed for three joint purposes: to prevent the CRISPR-Cas9 recutting after a successful edit; to create a primer binding site for sensitive PCR detection of edited genomic DNA; and to create an Apo1 restriction site for robust genotyping once a mutant allele was established (Fig. 1A,D-F). The intended edited sequence was checked through intron splicing predictive software to avoid inadvertently perturbing mRNA splicing.

Zebrafish embryos, at one-cell stage, were injected with a mix of the donor ssODN, Cas9 protein, generic trans-activating CRISPR RNA (tracrRNA) oligonucleotide and guide CRISPR RNA (crRNA) oligonucleotide. As a negative control, sibling embryos were left uninjected or injected with donor ssODN alone. At 3 days post fertilisation (3 dpf), some individual embryos were sacrificed and analysed by PCR with a primer specific for the intended genome edit, together with a primer external to the donor ssODN sequence region (Fig. 1A-D). Agarose gel electrophoresis revealed robust amplification of the expected 103 bp amplicon from each of the embryos injected with CRISPR-Cas9 and donor ssODN but not those with donor alone (Fig. 1D). In addition to the expected 103 bp amplicon, embryos injected with CRISPR-Cas9 and donor also gave multiple lower-mobility amplicons that differed between embryos. Reduced electrophoretic mobility is consistent with the indel types identified in previous reports of ssODN knock-in leading to integrated fragments of donor and partially mismatched hybrid amplicons (Boel et al., 2018; Prykhozhij et al., 2018). These injected embryos are interpreted as likely to be genetic mosaic, with a mix of unaltered cells, some cells with alleles involving indels together with the edit, and some cells with the intended edit (Boel et al., 2018; Prykhozhij et al., 2018).

Injected embryos (G0) were raised to adulthood, and nine adults were outcrossed to identify and isolate a line with the intended *pik3cd* edit. From each outcross, 24 embryos were lysed separately in individual wells in a 96-well plate. As a preliminary screen, the edit-specific PCR test was conducted on three pools of eight embryo lysates from each outcross. Lysates from positive pools were then retested individually (Fig. 1E), and, if positive, a 152 bp PCR product across the edit region was sequenced. When that sequence had the intended edit, overlapping 518 bp and 658 bp PCR products were sequenced, spanning the edit.

This analysis indicated that four of the nine tested G0 adults did not exhibit germline transmission of the *pik3cd* edit, three G0 adults transmitted edits combined with unwanted indels, and two G0 adults transmitted the intended *pik3cd^E525K^* edit. Embryos were raised from one of these fish, and *pik3cd^E525K^* adults were identified by edit-specific PCR from fin-clip lysates. Sequencing from PCR amplicons covering 891 bp across the edit region confirmed the integrity of the edit in the established line (Fig. 1B,G). This line has the allele designation *pik3cd^sh673^* but for clarity here will be referred to as *pik3cd^E525K^*.

When progeny from *pik3cd^E525K/+^* in-crosses were raised, no obvious external phenotype was apparent, and genotypes of the resulting adults followed the expected Mendelian ratio, with 20 *pik3cd^+/+^*, 31 *pik3cd^E525K/+^* and 16 *pik3cd^E525K/E525K^* (Chi-squared test, *P*=0.65).

### Homology-directed CRISPR-LbCpf1 knock-in of a zebrafish model for *PIK3CD^E1021K^*

**Fig. 2.**
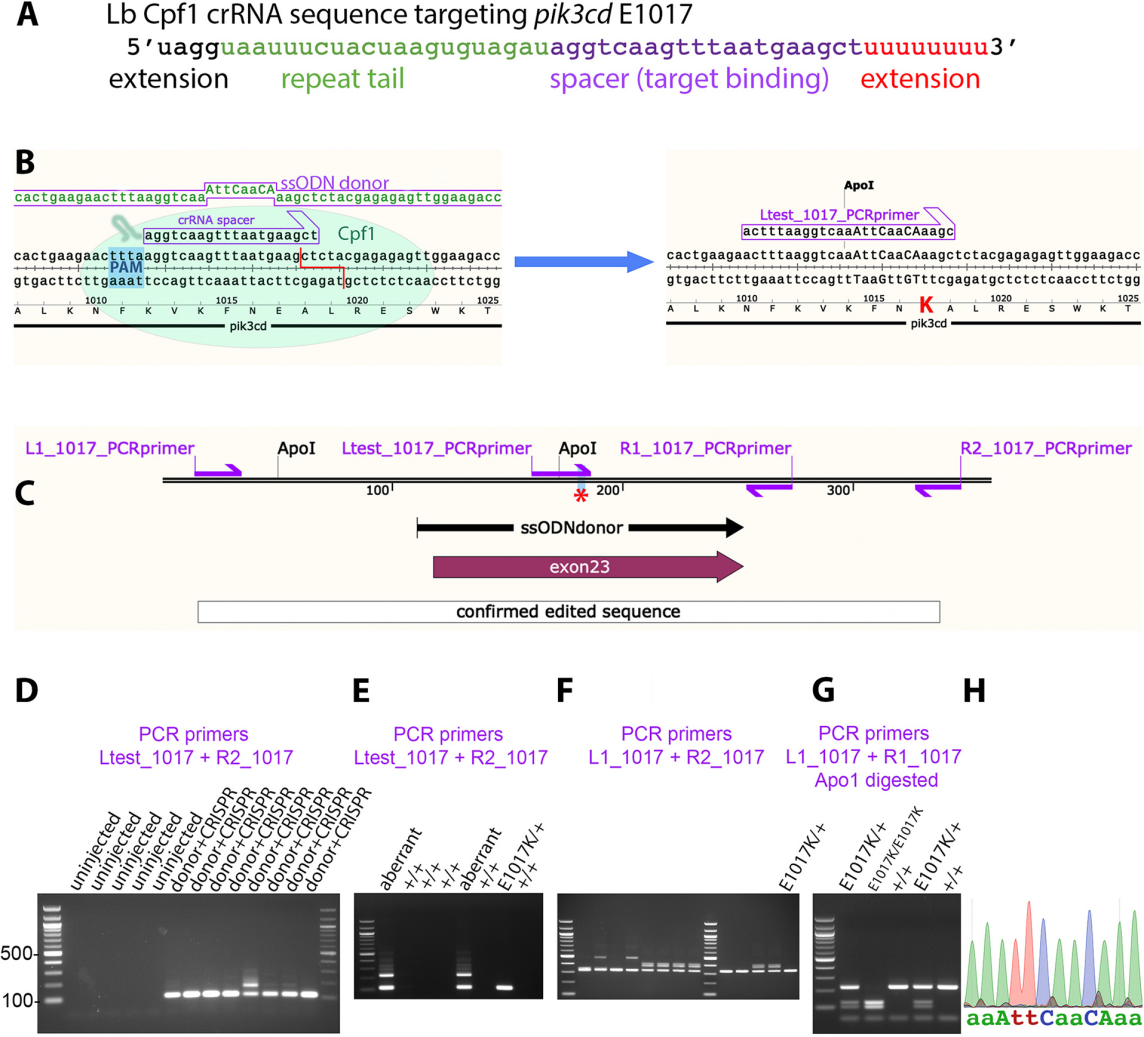
**Homology-directed CRISPR-LbCpf1 knock-in of a *pik3cd^E1017K^* allele.** (A) Sequence of crRNA oligonucleotide, showing 5′ and 3′ extensions beyond the minimal sequence of repeat tail and target binding spacer. (B) Diagram illustrating the knock-in process. The CRISPR-LbCpf1 cleavage and knock-in is illustrated on the left and the resulting *pik3cd^E1017K^* allele on the right. On the left, the ssODN donor sequence and crRNA spacer sequence are positioned alongside the *pik3cd* DNA sequence and translated amino acid sequence, with the CRISPR-LbCpf1 PAM highlighted in blue and the cleavage site shown as a red line. On the right, mutated DNA bases are shown in upper case, the E1017K coding change is highlighted in red, and the introduced ApoI cleavage site is indicated. The Ltest_1017 PCR primer is specific for the knock-in allele. (C) Map of the zebrafish *pik3cd^E1017K^* locus. PCR primers are shown as magenta arrows; the mutation site is marked with a red asterisk; and positions are shown for the ssODN donor used for knock-in, the region confirmed by sequencing after knock-in and *pik3cd* exon 23. (D) Agarose electrophoresis gel with knock-in-specific test PCRs from 3 dpf G0 embryos. All the embryos previously injected with CRISPR-LbCpf1 and ssODN donor produced the expected 113 bp amplicon and sometimes also lower electrophoretic mobility products. PCRs from uninjected embryos did not amplify. DNA size marker is 100 bp ladder. (E) Agarose electrophoresis gel with knock-in-specific test PCRs from 3 dpf F1 embryos from an outcross of a G0 adult that had been injected with CRISPR-LbCpf1 and ssODN donor when an embryo. A *pik3cd^E1017K/+^* embryo produced the 113 bp amplicon; other embryos produced lower-mobility amplicons indicating aberrant knock-in events. DNA size marker is 100 bp ladder. (F) Agarose electrophoresis gel with PCRs spanning the knock-in locus to further test embryos that had given the expected 113 bp amplicon with test PCRs as in E. The *pik3cd^E1017K/+^* embryo indicated in E gave the expected 332 bp amplicon, but some other embryos produced lower-mobility amplicons indicating aberrant knock-in events despite having given the expected 113 bp amplicon with test PCRs as in E. (G) Agarose electrophoresis gel with Apo1-digested PCR amplicons from F2 adults from a *pik3cd^E1017K/+^* in-cross. The *pik3cd^E1017K^* allele introduces an Apo1 site, allowing *pik3cd^E1017K/E1017K^*, *pik3cd^E1017K/+^* and *pik3cd^+/+^* genotypes to be distinguished. DNA size marker is 100 bp ladder. (H) Sanger sequencing trace from a PCR amplicon across the knock-in locus of a *pik3cd^E1017K/E1017K^* F2 adult. The mutated DNA bases are shown in upper case as in B.

Although the zebrafish *pik3cd* E1017 site (equivalent to the human *PIK3CD* E1021) lacks nearby potential CRISPR-Cas9 cleavage sites, it is 3 bp from a predicted CRISPR-LbCpf1 cleavage site. We made use of this alternative CRISPR technology for ssODN-directed gene editing. We broadly followed the method of Moreno-Mateos et al. (2017) and Fernandez et al. (2018), but with a crRNA guide oligonucleotide with 5′ and 3′ extensions (Bin Moon et al., 2018; Park et al., 2018) (Fig. 2A). A 142mer, symmetrical, non-template strand, knock-in donor ssODN was designed with two phosphothioates at each end. In addition to the point mutation causing E1017K, four additional silent point mutations were included (Fig. 2B). As with the E525K strategy, these were designed to prevent the CRISPR recutting and to facilitate allele identification while avoiding inadvertently perturbing mRNA splicing (Fig. 2B-E,G).

LbCpf1 protein was pre-incubated with guide crRNA before addition of donor ssODN and injected into one-cell-stage zebrafish embryos. At 3 dpf, some individual embryos were sacrificed and analysed by PCR with a primer specific for the intended genome edit, together with a primer external to the donor ssODN sequence region (Fig. 2B,C). Agarose gel electrophoresis revealed robust amplification of the expected 113 bp amplicon from each of the embryos injected with CRISPR-LbCpf1 and donor ssODN but not from uninjected controls (Fig. 2D). One of the injected embryos also gave an additional lower-mobility amplicon. As with the *pik3cd^E525K^* knock-in, these injected embryos are interpreted as likely to be genetic mosaic, containing some cells with alleles involving indels together with the edit.

Injected (G0) embryos were raised to adulthood, and 51 adults were outcrossed to identify and isolate a line with the intended *pik3cd* edit. From each outcross, 24 embryos were lysed separately in individual wells in a 96-well plate. As a preliminary screen, the edit-specific PCR test was conducted on three pools of eight embryo lysates from each outcross. Edit-specific PCR amplification was observed for 19 of the 51 G0 outcrosses. Lysates from positive pools were then retested individually, and, if positive, a 332 bp PCR product across the edit region was sequenced.

In some cases, either the edit-specific test PCR or the PCR spanning the edit region gave amplicons with lower-than-expected electrophoretic mobility (Fig. 2E,F). Additionally, some embryos identified with the allele-specific test PCR subsequently gave wild-type (wt) sequence from the 322 bp PCR product spanning the edit region. Nevertheless, we did identify one adult transmitting a *pik3cd^E1017K^* allele with the correct sequence across the 332 bp region (Fig. 2E). Two-hundred and fifty-four embryos from this fish were raised to adulthood, and 22 were identified as *pik3cd^E1017K^* by edit-specific PCR from fin-clip lysates. The 332 bp PCR amplicon across the edit region was sequenced to confirm the integrity of the edit in the established line (Fig. 2C,H). This line has the allele designation *pik3cd^sh674^* but for clarity here will be referred to as *pik3cd^E1017K^*.

When progeny from a *pik3cd^E1017K/+^* in-cross were raised, no obvious external phenotype was apparent, and genotypes of the resulting adults followed the expected Mendelian ratio, with seven *pik3cd^+/+^*, 13 *pik3cd^E1017K/+^* and six *pik3cd^E1017K/E1017K^* (Chi-squared test, *P*=0.96).

### *pik3cd^E1017K/+^* embryos and *pik3cd^E525K/+^* embryos exhibit increased neutrophilic inflammation following tail-fin injury

**Fig. 3.**
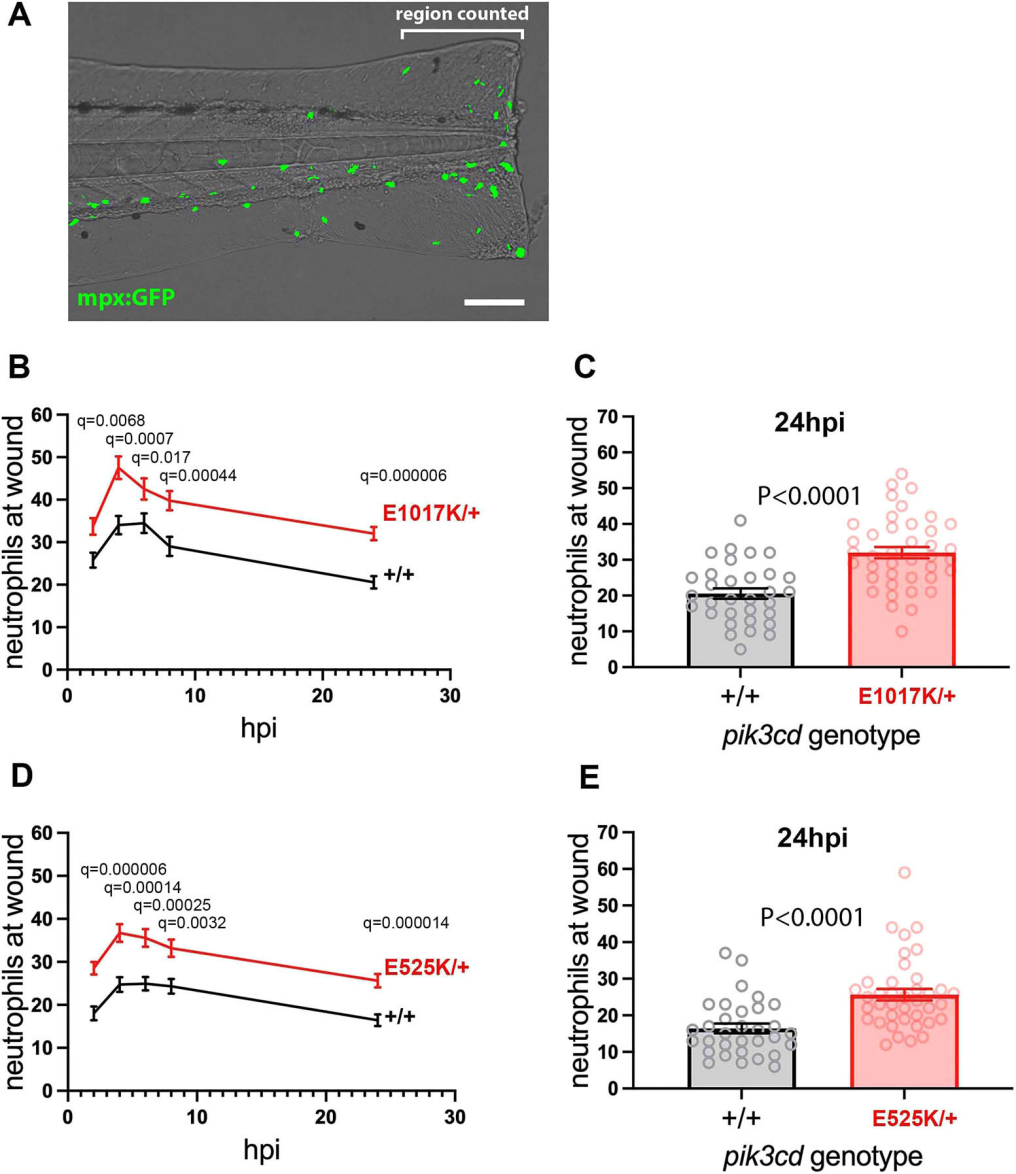
**Neutrophilic inflammation following tail-fin injury of *pik3cd^E1017K/+^* embryos and *pik3cd^E525K/+^* embryos.** (A) Composite brightfield and maximal projection of widefield fluorescent (shown in green) micrograph stack, illustrating the *TgBAC(mpx:gfp)i114* 3 dpf embryo tail-fin injury used to analyse inflammation response. Transection is through the tip of the notochord, and GFP marked neutrophils counted proximal to the circulatory loop. Scale bar: 100 µm. (B) Chart of the tail-fin injury inflammation time course for 72, 3 dpf embryos from a *pik3cd^E1017K/+^* outcross. Successive neutrophil counts at the injury site are shown as the mean±s.e.m. for embryos subsequently genotyped as *pik3cd^E1017K/+^* (red) or *pik3cd^+/+^* (black). Multiple Mann–Whitney tests. (C) Chart with the same data as in B but showing the neutrophil counts for the individual embryos at the 24 h post injury (hpi) end point of the analysis. Mann–Whitney test. Data shown are from one experiment. A repeat experiment with 71 embryos showed the same effect (*P*<0.0001 at 24 hpi), as did a 36-embryo, 4 hpi time point, pilot experiment (*P*=0.001). (D) Chart of the tail-fin injury inflammation time course for 71, 3 dpf embryos from a *pik3cd^E525K/+^* outcross. Successive neutrophil counts at the injury site are shown as the mean±s.e.m. for embryos subsequently genotyped as *pik3cd^E525K/+^* (red) or *pik3cd^+/+^* (black). Multiple Mann–Whitney tests. (E) Chart with the same data as in D but showing the neutrophil counts for the individual embryos at the 24 hpi end point of the analysis. Mann–Whitney test. Data shown are from one experiment. A repeat experiment with 96 embryos showed the same effect (*P*=0.0004 at 24 hpi).

Human patients with APDS1 are heterozygous for an activating *PIK3CD* mutant allele. We investigated whether our zebrafish *pik3cd* knock-in mutations similarly exhibited a phenotype when heterozygous with a wt *pik3cd* allele. We used an established embryo tail-fin injury assay to investigate whether either of our *pik3cd* knock-in mutants influenced the inflammatory response (Lieschke et al., 2001; Renshaw et al., 2006) (Fig. 3A).

Heterozygous carriers of the *pik3cd^E1017K^* or *pik3cd^E525K^* alleles were outcrossed to fish with the fluorescent neutrophil marker transgene *TgBAC(mpx:gfp)i114*. This allowed us to blindly compare sibling embryos that were either heterozygous for the *pik3cd* mutation or fully wt (in the expected Mendelian 1:1 ratio). At 3 dpf, embryos were injured, and fluorescent neutrophils were serially counted at the wound site from 2 h post injury (hpi) until 24 hpi, spanning the normal inflammation and resolution time course. The embryos were then genotyped by PCR across the *pik3cd* mutation site and Apo1 digestion.

Compared to their wt siblings, the embryos with either *pik3cd^E1017K/+^* or *pik3cd^E525K/+^* had increased neutrophil counts at the wound site, not only at the 4 hpi peak inflammatory time point but also throughout the time course (Fig. 3B-E).

### *pik3cd^E1017K/+^* embryos and *pik3cd^E525K/+^* embryos have increased total body neutrophils

**Fig. 4.**
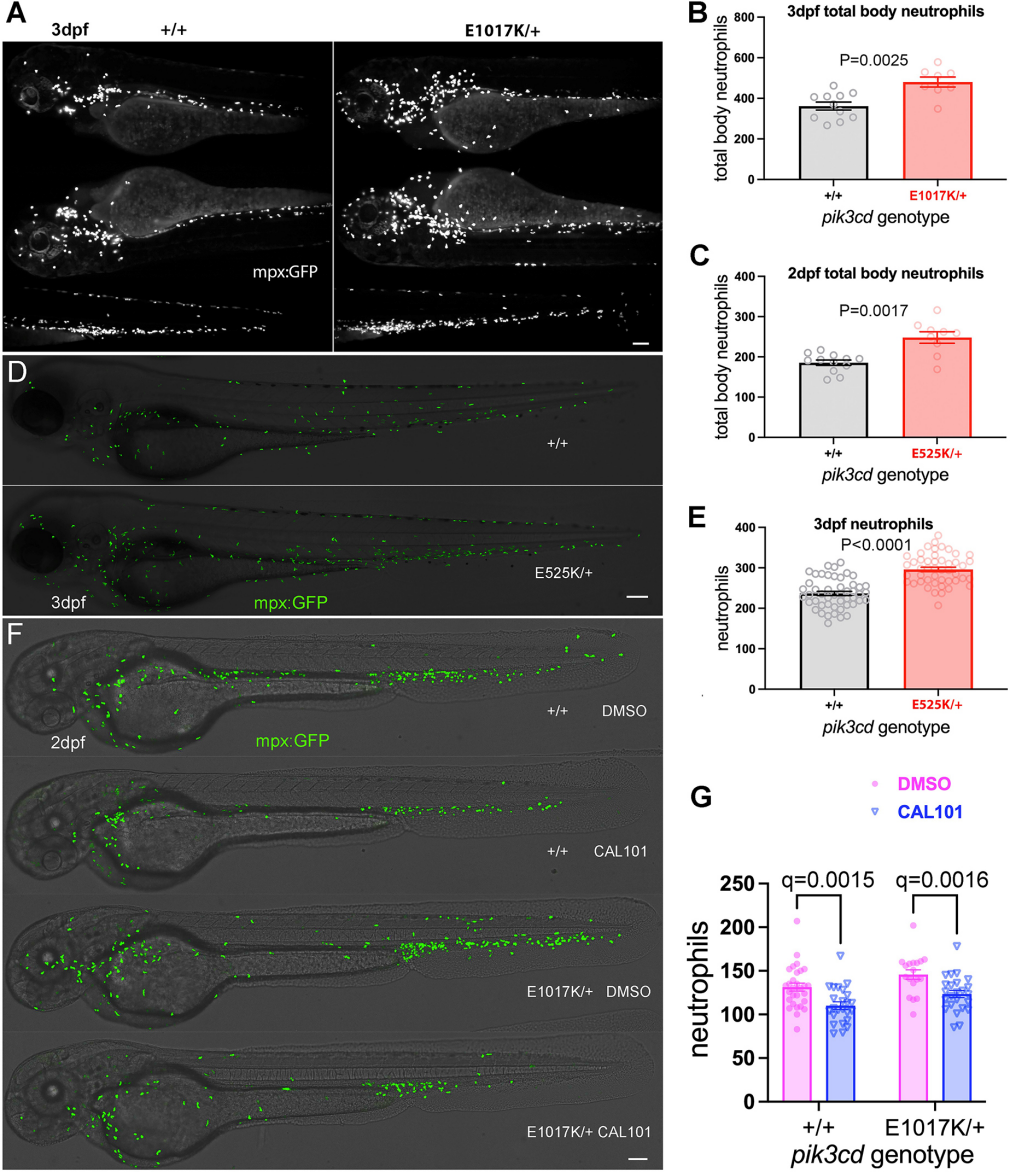
**Effect on total body neutrophils from *pik3cd^E1017K/+^* and *pik3cd^E525K/+^* genotype and the PI3Kδ inhibitor CAL-101.** (A) Light-sheet microscopy used to count total body *TgBAC(mpx:gfp)i114* neutrophils of *pik3cd^E1017K/+^* and *pik3cd^+/+^* 3 dpf, sibling embryos. Upper panels show maximal-intensity projections for the three views used for each embryo. Scale bar: 100 µm. (B) Chart of total body neutrophils counted from light-sheet microscopy views as in A for 19 sibling 2 dpf *pik3cd^E1017K/+^*_­_and *pik3cd^+/+^* embryos. Bars show the mean±s.e.m., Mann–Whitney test. Data shown are from one experiment. (C) Chart of total body neutrophils counted from light-sheet microscopy views as in A, but for 21 *pik3cd^E525K/+^*_­_and *pik3cd^+/+^* 2 dpf sibling embryos. Bars show the mean±s.e.m., Mann–Whitney test. Data shown are from one experiment. (D) Composites of brightfield and the maximal-intensity projections of spinning disk confocal fluorescent (shown in green) micrograph stacks used to count *TgBAC(mpx:gfp)i114* neutrophils in sibling *pik3cd^E525K/+^*_­_ and *pik3cd^+/+^* 3 dpf embryos. Scale bar: 100 µm. (E) Chart of neutrophils counted from single-sided maximal-intensity projections as in D for 96 sibling *pik3cd^E525K/+^*_­_ and *pik3cd^+/+^* 3 dpf embryos. Bars show the mean±s.e.m., Mann–Whitney test. Data shown are from one experiment. (F) Composites of brightfield and the maximal-intensity projections of spinning disk confocal fluorescent (shown in green) micrograph stacks used to count *TgBAC(mpx:gfp)i114* neutrophils in sibling *pik3cd^E1017K/+^* and *pik3cd^+/+^* 2 dpf embryos exposed between 24 hpf and 48 hpf to either 25 µM CAL-101 in 1% DMSO or a 1% DMSO vehicle control. Scale bar: 100 µm. (G) Chart of neutrophils counted from single-sided maximal-intensity projections as in F for 95 sibling *pik3cd^E1017K/+^* and *pik3cd^+/+^* 2 dpf embryos exposed between 24 hpf and 48 hpf to either 25 µM CAL-101 or a DMSO vehicle control. Bars show the mean±s.e.m., multiple Mann–Whitney tests. Data shown are from one experiment. Two pilot experiments with CAL-101 also showed reduced neutrophils.

Following our observations from the tail-fin injury assay, we sought to establish whether *pik3cd^E1017K/+^* and *pik3cd^E525K/+^* embryos simply had more neutrophils, even in the absence of an inflammatory stimulus. Sibling embryos from a *pik3cd^E1017K/+^* outcross with *TgBAC(mpx:gfp)i114* were analysed by light-sheet microscopy at 3 dpf to count total body neutrophils and then genotyped (Fig. 4A,B). Similarly, embryos from a *pik3cd^E525K/+^* outcross were analysed at 3 dpf with spinning disk microscopy and at 2 dpf with light-sheet microscopy (Fig. 4C-E). In each case, the embryos with *pik3cd* knock-in mutation had higher total body neutrophil numbers than their wt siblings.

We also investigated whether *pik3cd^E1017K/+^* adult zebrafish had an increased proportion of neutrophils in their whole-kidney marrow (WKM; the zebrafish adult haemopoietic tissue). Fish from *pik3cd^E525K/+^* outcrosses with *TgBAC(mpx:gfp)i114* were raised together until the age of 8 months. Flow cytometry of their WKM was used to identify leukocytes and precursors (Traver et al., 2003) and count the proportion from that cell population expressing the neutrophil marker *TgBAC(mpx:gfp)i114*. This analysis did not detect a significant difference between the *pik3cd^E525K/+^* adults and their wt siblings (Fig. S1).

### The PI3Kδ inhibitor CAL-101 reduces neutrophil numbers

Because the *pik3cd* knock-in mutations were expected to act by increasing PI3Kδ activity, we tested whether the neutrophilia phenotype was counteracted by exposure to the PI3Kδ-specific inhibitor CAL-101 (Idelalisib) (Lannutti et al., 2011). Sibling embryos from a *pik3cd^E1017K/+^* outcross with *TgBAC(mpx:gfp)i114* were exposed to CAL-101 from 24 h post fertilisation (hpf) until 48 hpf and then analysed by spinning disk microscopy and genotyped. CAL-101 exposure reduced neutrophil numbers in both the *pik3cd^E1017K/+^*embryos and their wt siblings (Fig. 4F,G).

### Excessive neutrophilic inflammation is also observed after the definitive wave and the neutrophils are differentiated

The neutrophilia we observed with our *pik3cd* knock-in mutants suggested comparison with previous findings from zebrafish lacking Pten. It has previously been shown that *ptena^−/−^;ptenb^−/−^* double mutants have excessive *mpx*-expressing neutrophils, both from the primitive and the definitive wave. However, the *ptena^−/−^;ptenb^−/−^* definitive wave neutrophils fail to fully differentiate and so, at 5 dpf, a large proportion of tail neutrophils are not stainable with Sudan Black dye (Choorapoikayil et al., 2014).

**Fig. 5.**
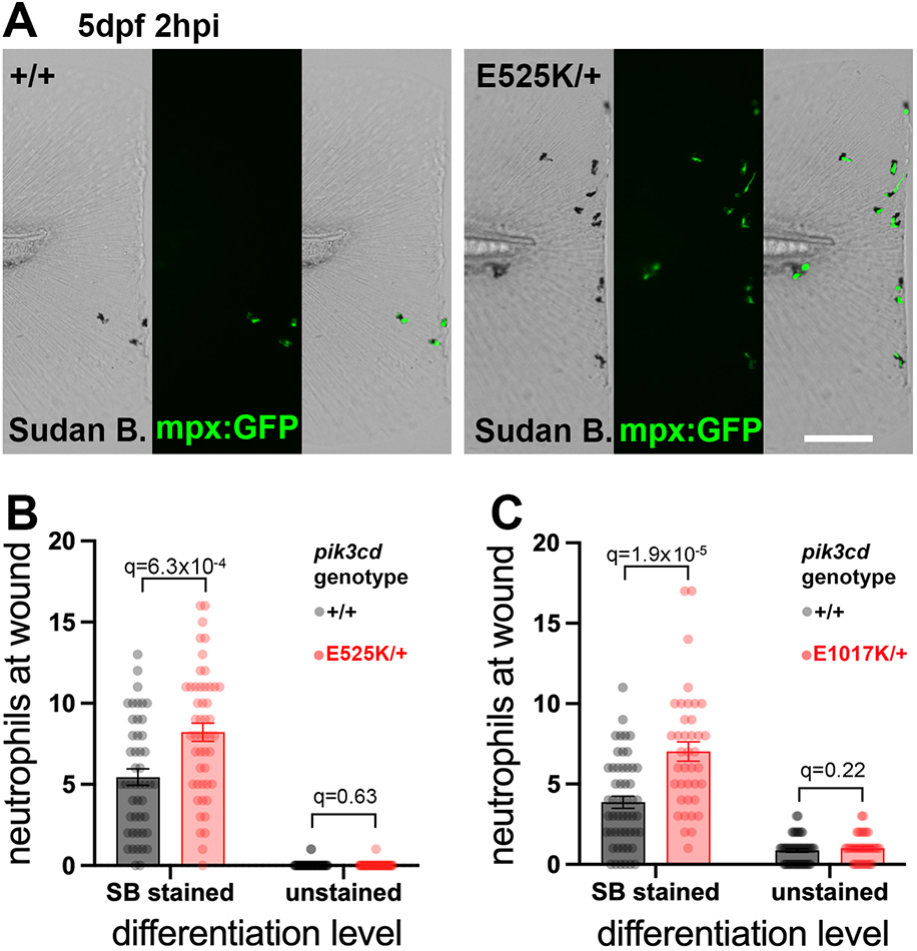
**Sudan Black assessment of neutrophil differentiation at tail-fin injury site at 5** **dpf.** (A) Brightfield and maximal-intensity projection of widefield fluorescent micrographs of tail fins of Sudan Black (SB)-stained, 5 dpf, *TgBAC(mpx:gfp)i114*; *pik3cd^E525K/+^*_­_and *pik3cd^+/+^* sibling embryos fixed 2 hpi after transection of the tip of the tail fin. Scale bar: 100 µm. (B) Chart of neutrophil counts at tail-fin injury site, from 96 sibling 5 dpf, *pik3cd^E525K/+^*_­_and *pik3cd^+/+^* embryos as in A. Counts are shown both for Sudan Black-stained neutrophils and for unstained neutrophils marked by *TgBAC(mpx:gfp)i114*. Bars show the mean±s.e.m., multiple Mann–Whitney tests. Data shown are from one experiment. A pilot experiment with *pik3cd^E525K/+^* also showed that *TgBAC(mpx:gfp)i114*-marked cells were almost all Sudan Black stained. (C) Chart of neutrophil counts at tail-fin injury site, from 92 sibling 5 dpf, *pik3cd^E1017K/+^*_­_and *pik3cd^+/+^* embryos. Counts are shown both for Sudan Black-stained neutrophils and for unstained neutrophils marked by *TgBAC(mpx:gfp)i114*. Bars show the mean±s.e.m., multiple Mann–Whitney tests. Data shown are from one experiment.

We used a 5 dpf tail-fin injury assay to analyse definitive wave neutrophils in *pik3cd^E1017K/+^* and *pik3cd^E525K/+^* larvae. Sibling larvae from a *pik3cd^E1017K/+^* or *pik3cd^E525K/+^* outcross with *TgBAC(mpx:gfp)i114* were subjected to a minor tail-fin injury at 5 dpf. The larvae were fixed at 2 hpi, stained with Sudan Black dye, and imaged to identify Sudan Black-stained and *TgBAC(mpx:gfp)i114* neutrophils at the wound region (Fig. 5A). Genotyping, after analysis, showed that the mutant larvae had excessive Sudan Black-stained neutrophils at the wound. Irrespective of *pik3cd* genotype, there were very few *TgBAC(mpx:gfp)i114* neutrophils not stained with Sudan Black (Fig. 5B,C).

### *pik3cd^E1017K/+^* embryos have unaltered total body macrophage counts and unaltered susceptibility to a *Staphylococcus aureus* infection model

**Fig. 6.**
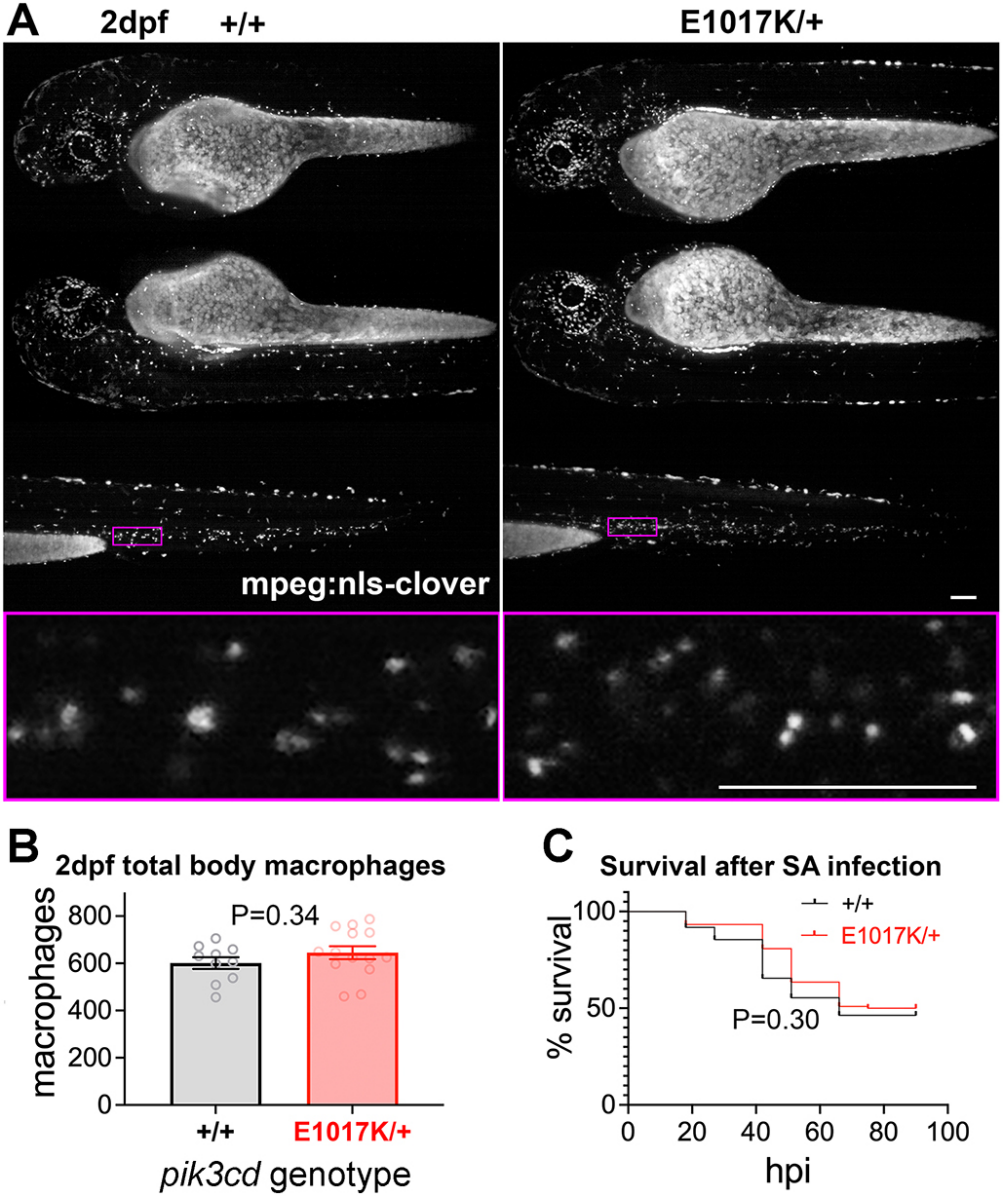
**Total body macrophages in *pik3cd^E1017K/+^* embryos and survival following *S. aureus* infection.** (A) Light-sheet microscopy used to count total body *Tg(mpeg1.1:NLS-clover)sh616* macrophages of *pik3cd^E1017K/+^* and *pik3cd^+/+^* 2 dpf, sibling embryos. Top three rows show maximal-intensity projections for the three views used for each embryo. The magenta border shows the region displayed in the bottom row as a single-slice view from the image stack at higher magnification as used for counting. Scale bars: 100µm. (B) Chart of total body macrophages counted from light-sheet microscopy views as in A for 24 sibling 2 dpf *pik3cd^E1017K/+^*_­_and *pik3cd^+/+^* embryos. Bars show the mean±s.e.m., Mann–Whitney test. Data shown are from one experiment. (C) Survival of 214 sibling *pik3cd^E1017K/+^*_­_and *pik3cd^+/+^* embryos following injection of 1500 colony-forming units of *S. aureus* (SA) into the circulation. Log-rank (Mantel–Cox) test. Data shown are from one experiment. A repeat experiment with 229 embryos showed similar results (*P*=0.99 between genotypes).

We investigated whether the observed increase in total body neutrophils was accompanied by any alteration in total body macrophage numbers. We used the fluorescent macrophage nuclear marker transgene *Tg(mpeg1.1:NLS-clover)sh616*. Sibling embryos from a *pik3cd^E1017K/+^* outcross with *Tg(mpeg1.1:NLS-clover)sh616* were analysed by light-sheet microscopy at 2 dpf to count total body macrophages and then genotyped. We observed no difference in the total body macrophage numbers between the *pik3cd^E1017K/+^* embryos and their wt siblings (Fig. 6A,B).

Innate immune cell function is known to be critical for resistance to the pathogen *S. aureus* in the circulation of larval zebrafish (Prajsnar et al., 2008, 2012). We tested whether the *pik3cd^E1017K/+^* mutation altered susceptibility to such infection. Sibling 30 hpf embryos from a *pik3cd^E1017K/+^* outcross were intravenously injected with 1500 colony-forming units of *S. aureus*. Dead and dying embryos were collected over the following 90 h and then genotyped, along with all those still surviving at the end. The survival of the *pik3cd^E1017K/+^* larvae was unaltered from that of their wt siblings, with the expected ∼50% mortality (Fig. 6C).

### *pik3cd^E1017K/+^* and *pik3cd^E525K/+^* embryos have normal tail-fin regeneration

**Fig. 7.**
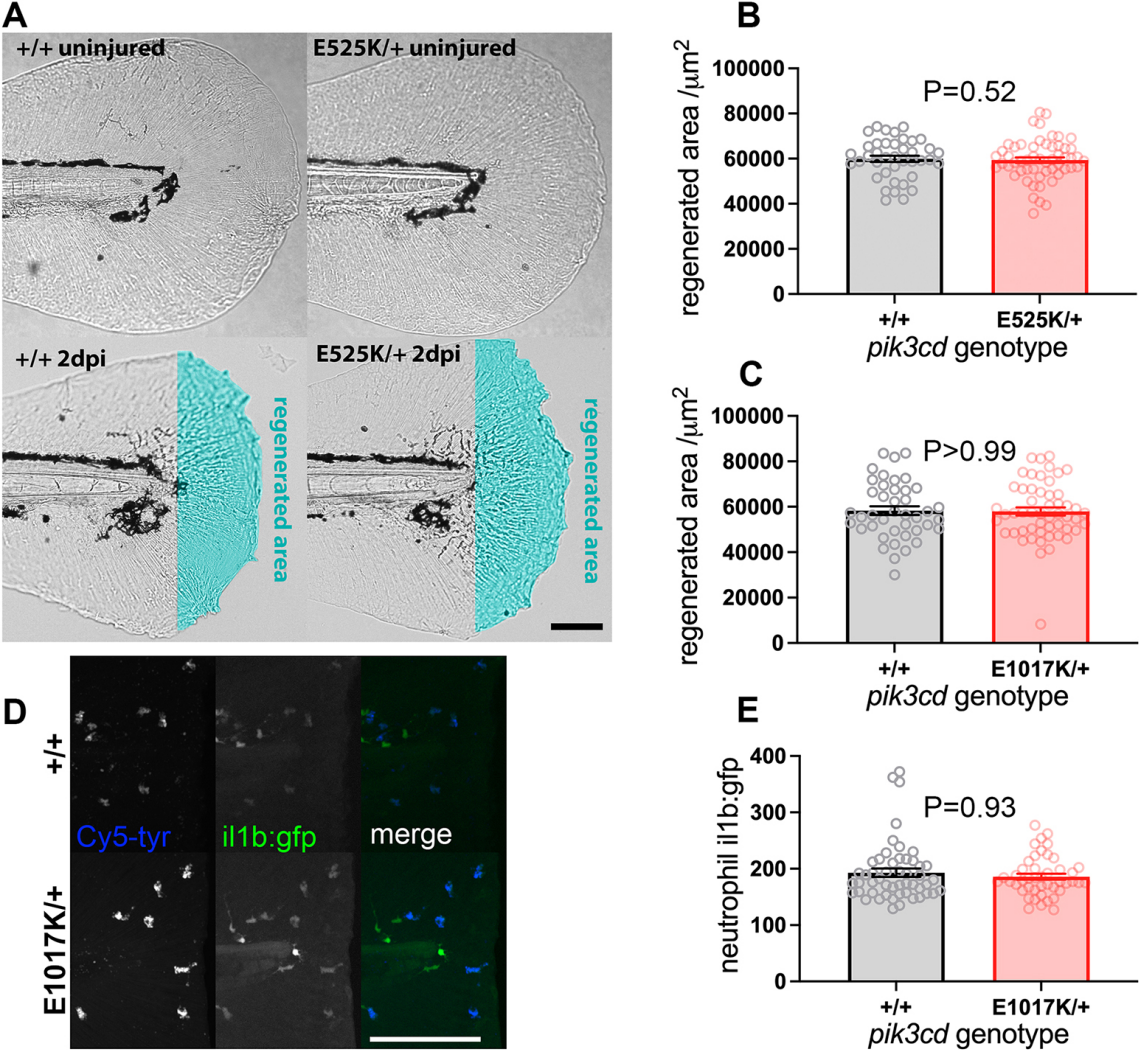
**Tail-fin regeneration in *pik3cd^E525K/+^* embryos.** (A) Brightfield micrographs used to measure tail-fin regeneration for *pik3cd^E525K/+^* embryos and their *pik3cd^+/+^* siblings at 5 dpf, 2 days post injury (dpi). Top row shows uninjured controls. Transection was through the tip of the notochord. The regenerated area is illustrated with cyan false colour. Scale bar: 100 µm. (B) Chart of tail-fin regenerated area from micrographs as in A for 96 embryos. Bars show the mean±s.e.m., Mann–Whitney test. Data shown are from one experiment. A similar experiment using 5 dpf, 3 dpi larvae also detected no effect. (C) Chart of tail-fin regenerated area for *pik3cd^E1017K/+^* embryos and their *pik3cd^+/+^* siblings at 5 dpf, 2 dpi. Measured from micrographs of 96 embryos. Bars show the mean±s.e.m., Mann–Whitney test. Data shown are from one experiment. (D) Maximal-intensity projections of spinning disk confocal fluorescent micrograph stacks of 3 dpf *TgBAC(il1b:EGFP)sh445*;*pik3cd^E1017K/+^* embryos and their *pik3cd^+/+^* siblings, fixed and stained 3 h after tail transection. Fluorescence channels are shown for GFP and for endogenous peroxidase activity stain with Cy5-tyramide to identify neutrophils*.* Scale bar: 100 µm. (E) Chart of neutrophil *TgBAC(il1b:EGFP)sh445* fluorescence intensity for single neutrophils at the injury site from each of 96 3 dpf sibling *pik3cd^E1017K/+^* and *pik3cd^+/+^* larvae, fixed and stained 3 h after tail transection as in D. Bars show the mean±s.e.m., Mann–Whitney test. Data shown are from one experiment.

In some contexts, excessive neutrophilic inflammation is implicated in impaired regeneration after injury (Tsarouchas et al., 2018; Bernut et al., 2020). We investigated whether there was also such an effect from the excessive neutrophilic inflammation we observed after tail-fin injury in our *pik3cd* knock-in mutants. At 3 dpf, tail fins of *pik3cd^E1017K/+^* or *pik3cd^E525K/+^* embryos and their wt siblings were injured and then allowed to regenerate for 2 days before imaging, measurement and genotyping. Equally extensive areas of tail fin regenerated in the mutant embryos and their wt siblings (Fig. 7A-C).

Neutrophil Il-1β plays an important role in mediating the influence of neutrophilic inflammation on regeneration (Tsarouchas et al., 2018). The *TgBAC(il1b:EGFP)sh445* reporter has previously been shown to be transiently upregulated in neutrophils after a 3 dpf tail-fin injury (Ogryzko et al., 2019). We used this assay to compare expression levels between *pik3cd^E1017K/+^* embryos and their wt siblings. Embryos were fixed 3 h after injury and stained for myeloperoxidase activity to identify neutrophils. Using spinning disk microscopy, GFP fluorescence was measured in neutrophils, and the embryos were genotyped. This analysis indicated that each neutrophil in *pik3cd^E1017K/+^* embryos had similar Il-1β expression to that in the wt siblings (Fig. 7D,E).

### *pik3cd^E1017K/+^* embryos have normal neutrophil migration

**Fig. 8.**
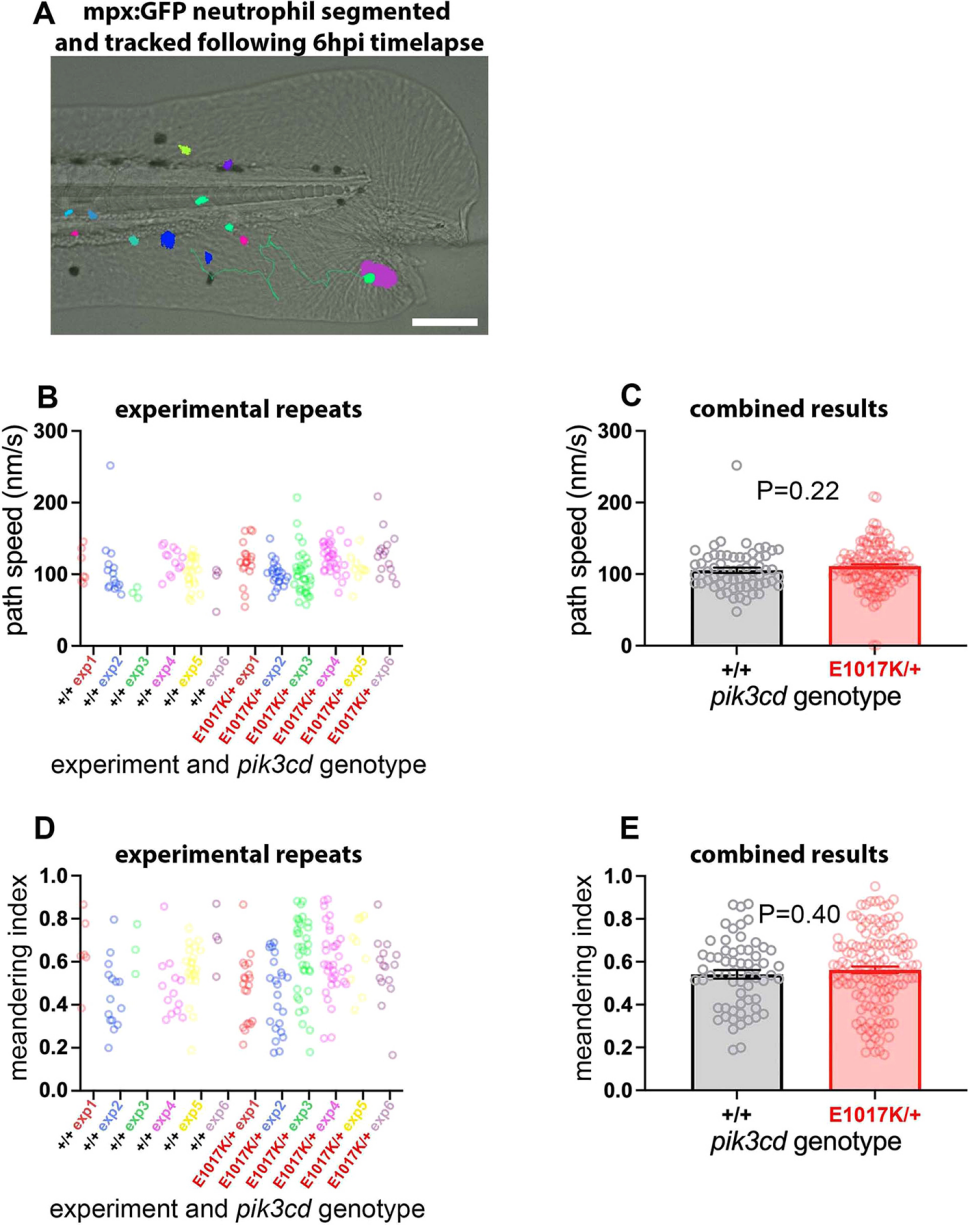
**Neutrophil migration in *pik3cd^E1017K/+^* embryos.** (A) Illustrative example of the *TgBAC(mpx:gfp)i114* tail-fin injury inflammation model used to track neutrophil migration at 3 dpf. Following a minor tail-fin injury and widefield time-lapse microscopy for 6 hpi, semi-automated segmentation and tracking with NIS Elements software determined migration paths to the injury site. Segmented *TgBAC(mpx:gfp)i114* neutrophils are shown as false-coloured patches, and a migration track is shown as a green line, superimposed on a brightfield micrograph (taken at the start of the time-lapse sequence). Scale bar: 100 µm. (B) Chart of path speeds for tracked neutrophils from sibling *pik3cd^E1017K/+^* and *pik3cd^+/+^* embryos as in A. Data are shown for each of six experiments, each with eight or nine embryos. (C) Chart of pooled data from B. Bars show the mean±s.e.m., unpaired *t*-test with Welch's correction. (D) Chart of meandering index (displacement/path length) for the tracked neutrophils as in B. (E) Chart of pooled data from D. Bars show the mean±s.e.m., unpaired *t*-test with Welch's correction.

PI3K signalling is known to direct neutrophil migration (Liu et al., 2007; Nishio et al., 2007; Yoo et al., 2010), and inhibition of PI3Kδ or PI3Kγ restored migratory accuracy exhibited by neutrophils from healthy elder volunteers and chronic obstructive pulmonary disease (COPD) patients, linking increased constitutive PI3K activity with inaccurate neutrophil migration and potentially enhanced bystander damage (Sapey et al., 2011, 2014). This raised the possibility that our *pik3cd* knock-in mutants might exhibit aberrant neutrophil migration. We used a *TgBAC(mpx:gfp)i114* tail-fin injury inflammation model to track neutrophil migration in 3 dpf *pik3cd^E1017K/+^* embryos and their wt siblings. Widefield time-lapse video microscopy allowed neutrophil migration to be tracked over a 6 h time course following a minor tail-fin needle injury (Fig. 8A). Videos of neutrophils that migrated to the injury site were used to analyse the neutrophil migration paths, and the embryos were genotyped. Consistent with our other observations, there were more neutrophils in the *pik3cd^E1017K/+^* embryos than in their wt siblings. However, no difference was detected for the neutrophil migration path speeds or meandering index of the two genotypes (Fig. 8B-E).

## DISCUSSION

Zebrafish gene editing to recapitulate human disease alleles has recently become an accessible approach, thanks to CRISPR and ssODN technology (Prykhozhij and Berman, 2018). CRISPR-Cas9 and CRISPR-LbCpf1 have differing DNA sequence requirements, which widens the scope of this method (Moreno-Mateos et al., 2017; Bin Moon et al., 2018). We had a favourable experience in using Cas9 to generate the *pik3cd^E525K^* allele and LbCpf1 to generate the *pik3cd^E1017K^* allele and would recommend either technology, depending on the DNA target site.

Our priority was to construct our *pik3cd* knock-in lines. During the procedure, we did not investigate the numerous instances in which preliminary analysis of putative edited alleles failed to confirm a correct edit. The fish we used for mutagenesis and screening were not pre-screened to ensure that they were isogenic around the targeting site. Consequently, pre-existing polymorphisms that interfere with PCR or sequencing may have caused us to miss some correctly edited alleles. The underlying knock-in efficiency could therefore have been higher than the overall efficiency we achieved. Using a stock of fish isogenic at the edit region might be preferable for any future gene-editing projects. It is also difficult to use our results to compare the relative editing efficiency of Cas9 and LbCpf1. However, both proved to be adequate for convenient use.

This study used embryos from outcrosses of fish heterozygous for the *pik3cd* knock-in mutations, and then genotyped the embryos after analysis. That enabled internally controlled comparison between mutant and wt sibling embryos, and avoided potential observer bias. For future work requiring bulk analysis or rapid screening, it may be more convenient to use clutches entirely of mutant embryos from a homozygous parent.

Human APDS1 patients are heterozygous for activating *PIK3CD* alleles, as are our zebrafish models using activating *pik3cd* alleles. Both our *pik3cd* knock-in alleles exhibited the same dominant neutrophilia phenotype. The only correlate for this that we are aware of from human APDS1 patients is a single case of bone marrow granulomatous hyperplasia (Lu et al., 2021). Previous studies of human APDS1 patients and mouse *Pik3cd^E1020K/+^* models did not report neutrophilia (Angulo et al., 2013; Lucas et al., 2014; Avery et al., 2018; Stark et al., 2018). It is possible that if there is a comparable effect from activated *pik3cd* mutations in mammals, it might be obscured by a confounding influence of adaptive immune system defects. Also, the small size and transparency of zebrafish embryos facilitates total body examination of neutrophils and macrophages, whereas mammalian investigations typically analyse blood, which may not always reflect total body cell numbers. These same caveats apply to our analysis of adult zebrafish marrow that similarly did not detect an effect from the activated *pik3cd* mutation. Potentially though, the mechanism causing the neutrophilia in larvae could be developmental-stage specific.

The neutrophilia phenotype of our knock-in alleles was counteracted by the PI3Kδ inhibitor CAL-101 (Idelalisib), which also reduced neutrophil numbers in wt embryos. Neutropenia is a common side effect for human patients treated with Idelalisib for haematological malignancies (de Weerdt et al., 2017). The identical phenotype observed in both *pik3cd* APDS1 knock-in alleles, plus the reversal with a clinical PI3Kδ inhibitor, gives confidence that the neutrophilia is indeed due to PI3Kδ hyperactivation, with potential relevance to the human disease.

The neutrophilia phenotype of our knock-in alleles is consistent with mechanisms for emergency granulopoiesis in mice (Kwak et al., 2015; Mistry et al., 2019). Emergency granulopoiesis generates extra neutrophils in response to inflammatory stimuli such as bacterial infection or experimental exposure to bacterial lipopolysaccharide. In response to this inflammatory stimulus, PI3Kδ-dependent AKT phosphorylation, in haemopoietic stem cells and bone marrow stromal cells, causes proliferation of progenitors and rapid differentiation to neutrophils (Kwak et al., 2015; Mistry et al., 2019). Emergency granulopoiesis has also been described in zebrafish embryos in which bacterial infection causes neutrophilia without an accompanying increase in macrophages (Hall et al., 2012). We observe that same pattern with our *pik3cd* knock-in alleles. Together, these findings may indicate that our *pik3cd* knock-in alleles constitutively induce a state analogous to emergency granulopoiesis.

The role of PI3Kδ during emergency granulopoiesis invites comparison to the role of PI3Kδ in acute myeloid leukaemia (AML). AML is an uncontrolled proliferation of abnormal myeloid progenitors. Intriguingly, although human AML blasts exhibit constitutive PI3Kδ activity, AML is not associated with *PIK3CD* mutations (Cornillet-Lefebvre et al., 2006; Darici et al., 2020). Mice with HSPCs lacking PTEN (Yilmaz et al., 2006; Zhang et al., 2006), or with hyperactive ATK (Kharas et al., 2010), develop AML. Zebrafish larvae lacking Pten similarly exhibit an expansion of HSPCs, and, although definitive myeloid and lymphoid lineages enter early stages of development, differentiation fails to complete (Choorapoikayil et al., 2014).

There are notable contrasts between our *pik3cd* knock-in mutants and zebrafish lacking Pten. Zebrafish lacking Pten are not viable and are deformed by 5 dpf, whereas our *pik3cd* knock-in mutants are adult viable. Zebrafish lacking Pten (owing to *ptena^−/−^;ptenb^−/−^* mutations) have excessive fully differentiated neutrophils from the primitive wave, whereas definitive wave neutrophils differentiate to the point of expressing *mpx* but not to having granules that can be stained with Sudan Black dye (Choorapoikayil et al., 2014). Consequently, at 5 dpf, zebrafish lacking Pten have excessive Sudan Black-stainable neutrophils in anterior regions but a pronounced deficit in the tail (Choorapoikayil et al., 2014). By contrast, our *pik3cd* knock-in mutants exhibited excessive neutrophilic inflammation in the tail at 5 dpf, and the neutrophils showed no reduction in Sudan Black staining. Presumably our knock-in mutants had sufficient PI3K signalling to share the *ptena^−/−^;ptenb^−/−^* phenotype of expanded early steps of myelopoiesis, but not so much as to stall later differentiation. The more subtle and restricted phenotypes of our *pik3cd* knock-in mutants offer further opportunities for investigating the role of PI3K signalling in haemopoiesis.

Recurrent bacterial respiratory infections are a feature of APDS1. Studies using genetic mosaic *Pik3cd^E1020K/+^* mice found that increased susceptibility to *S. pneumoniae* infection was attributable to lymphocyte defects (Stark et al., 2018). Zebrafish larvae provide an infection model dependent on macrophages and neutrophils, prior to the development of functional lymphocytes (Prajsnar et al., 2008, 2012). We found that *pik3cd^E1017K/+^* larvae had unaltered susceptibility to the *S. aureus* circulation infection model. This suggests that their macrophages and neutrophils retain bactericidal capability, in accord with the previous mouse findings.

Despite having excessive neutrophilic inflammation, our *pik3cd^E525K/+^* embryos were not defective in tail-fin regeneration after injury. This contrasts with other examples in which neutrophilic inflammation is associated with reduced regeneration. Zebrafish *runx1^−/−^* mutants have reduced neutrophils and accelerated regeneration after tail-fin injury (Li et al., 2012). Similarly, neutrophil-depleted mice have accelerated wound closure (Dovi et al., 2003). In a zebrafish larval spinal cord injury model, Il-1β produced by neutrophils impeded regeneration (Tsarouchas et al., 2018). We observed that the fluorescence intensity of the *TgBAC(il1b:EGFP)sh445* reporter in each neutrophil appeared as strong in *pik3cd^E1017K/+^* embryos, indicating that the neutrophils retained that inflammatory function in the mutant. The increased neutrophilic inflammation in our *pik3cd^E525K/+^* and *pik3cd^E1017K/+^* embryos after tail-fin injury was at a similar level to that reported for zebrafish *cystic fibrosis transmembrane conductance regulator* (*cftr^−/−^*) mutant embryos (Bernut et al., 2020). However, *cftr^−/−^* mutant embryos exhibit a pronounced reduction in tail-fin regeneration, alleviated by reducing neutrophils (Bernut et al., 2020). Differences in the regenerating tissue and/or the activation state of the neutrophils may account for this difference in regeneration between *cftr^−/−^* and our activating *pik3cd* mutations*.*

PI3K activity is a key determinant of neutrophil motility (Sasaki et al., 2000; Liu et al., 2007; Nishio et al., 2007; Yoo et al., 2010). Excessive PI3K activity in human neutrophils (from COPD patients or healthy volunteers age >65) is associated with defective chemotactic directionality (Sapey et al., 2011; Sapey et al., 2014). We considered it plausible that aberrant PI3Kδ might interfere with neutrophil migration in our *pik3cd^E1017K/+^* embryos. However, they exhibited apparently normal neutrophil motility in response to a tail-fin injury. Presumably, the required localised balance between PI3 kinase and phosphatase activities is still maintained to allow normal migration. Alternatively, a subtle defect may have been beyond the sensitivity of our assay.

It is not immediately obvious whether the neutrophilia phenotype of our knock-in mutants relates to the disease aetiology of APDS in human patients. However, insights into PI3Kδ signalling, with medical relevance beyond treatment of APDS itself, have come from the investigations into APDS mutations in humans and mice. The zebrafish knock-in mutations, described here, add significantly to this, and provide a model system suitable for further studies.

## MATERIALS AND METHODS

### Zebrafish strains, maintenance and pharmacological treatment

Zebrafish were maintained in accordance with UK Home Office regulations. Embryos were obtained by pair mating and incubated at 28°C in E3 medium (without Methylene Blue if used for imaging or infection assays). *TgBAC(mpx:gfp)i114* has previously been described (Renshaw et al., 2006). *Tg(mpeg1.1:NLS-clover)sh616* was isolated by outcrossing from the *Tg(mpeg1.1:NLS-clover)* stock described in Bernut et al. (2019). *Tg(mpeg1.1:NLS-clover)sh616* exhibits Mendelian transmission, indicating that it is a single insertion. To aid imaging for some experiments, embryos were either exposed to 200 µM 1-phenyl-2-thiourea from 24 hpf or had the *mitfa ^w2^* melanophore deficiency (Lister et al., 1999). Anaesthesia to allow handling, inspection or imaging used immersion in 0.164 mg/ml tricaine.

For CAL-101 exposure, dechorionated 24 hpf embryos were immersed in 25 µM CAL-101 (Selleckchem) in 1% dimethyl sulfoxide (DMSO) in E3 medium at 25 embryos per 8 ml volume glass Petri dish. To make the immersion mixture, CAL-101 dissolved in DMSO was added to vortexing E3 medium in a glass tube. Controls were similarly immersed in 1% DMSO in E3 medium.

### CRISPR ssODN directed knock-in

NNSPLICE 0.9 (Reese et al., 1997), ESEfinder (Smith et al., 2006) and Human Splicing Finder (Desmet et al., 2009) were used to screen against perturbation of predicted splicing when designing knock-in mutations. Knock-in of the *pik3cd^E525K^* allele followed Prykhozhij et al. (2018). A CRISPR-Cas9 target site was identified using Sequence Scan for CRISPR (Xu et al., 2015) and CHOPCHOP (Labun et al., 2016). One-cell-stage embryos were injected with 1 nl of a mix of 1 µM custom phosphorothioated (*) Ultramer oligonucleotide 5′-a*g*gtctttgcttctaaaataatggtttgttgatggattgaattgttgtagcaccagaagctgaaagagattgtggacaataaaaactccaccgaattcttcaaggatgagaaggagctgttgtggaagctgcgagagga*g*-3′ (IDT), 2 µM Cas9 Nuclease *S. pyogenes* (NEB), 20 µM tracrRNA (Merck), 20 µM custom crRNA (with spacer 5′-cucauccucaaagaacucgg-3′) (Merck) and 0.05% Phenol Red (Merck).

Knock-in of the *pik3cd^E1017K^* allele broadly followed Bin Moon et al. (2018), Fernandez et al. (2018) and Park et al. (2018). The CRISPR-Cpf1target site was identified using DeepCpf1 (Kim et al., 2018). Then, 1 µl of 200 µM custom RNA oligonucleotide 5′-uagguaauuucuacuaaguguagauaggtcaagtttaatgaagctuuuuuuuu-3′ (Merck), 0.2 µl of 10× NEB buffer 2.1 and 0.5 µl of 100 µM Lba Cas12a (Cpf1) (NEB) were mixed and incubated for 10 min at 25°C, before being added to 0.3 µl 0.5% Phenol Red (Merck) and 1 µl of 3 µM custom phosphorothioated (*) Ultramer oligonucleotide 5′-g*t*cacaggactctttggcactggggaaatcggaggaggaagcactgaagaactttaaggtcaaattcaacaaagctctacgagagagttggaagaccaaggtgaactggatgatgcacacatttgccaaagataacagata*a*-3′ (IDT). One-cell-stage embryos were injected with 1 nl of the resulting mix.

### PCR, genotyping and sequencing

PCR primers were designed with Primer3 (Untergasser et al., 2012). SnapGene software was used for molecular biology strategy and analysis.

Each 20 µl PCR reaction used 1 µl embryo lysate or 0.5 µl adult fin-clip lysate (prepared by incubation for 30 min at 98°C in 50 µl of 25 mM KOH, 200 µM EDTA, vortexing, neutralisation by addition of 50 µl of 40 mM Tris-HCl and centrifugation to precipitate debris). PCRs used FIREPol Master Mix (Solis BioDyne) with touch-down temperature cycling: 2 min at 94°C; ten cycles of [20 s at 94°C, 30 s at 60°C to 55°C (dropping 0.5°C per cycle), 45 s at 72°C]; 25 cycles of (20 s at 94°C, 30 s at 55°C, 45 s at 72°C); and 3 min at 72°C.

Screening for *pik3cd^E525K^* knock-in events used primers Ltest525 5′-aaaactccaccgaattcttcaag-3′ and R1_525 5′-atgagcaggagtttggggag-3′. Tests for the established *pik3cd^E525K^* allele used primers L2_525 5′-tggattgaattgttgtagcacca-3′ and R1_525 5′-atgagcaggagtttggggag-3′, followed by ApoI cleavage of the *pik3cd^E525K^* sequence. Sequence analysis of *pik3cd^E525K^* knock-in events was direct Sanger sequencing of PCR products from primers L2_525 with R1_525, or L3_525 5′-gaattgttgtagcaccagaagc-3′ with R3_525 5′-tgcacaaagagtaaacagcaca-3′, or L1_525 5′-tggggctattgtgttgcatt-3′ with R2_525 5′-tcacctgagccacatcctc-3′, using the PCR primers as sequencing primers.

Screening for *pik3cd^E1017K^* knock-in events used primers Ltest_1017 5′-actttaaggtcaaattcaacaaagc-3′ and R1_1017 5′-cagtctgtgatcgagcgttg-3′. Tests for the established *pik3cd^E1017K^* allele used primers L1_1017 5′-tgtcattggctgttcttgct-3′ and R1_1017, followed by ApoI cleavage of the *pik3cd^E1017K^* sequence. The primer L2_1017 5′-tcacaggactctttggcact-3′ was used with R1_1017 for genotyping degraded larvae following Staphylococcus infection. Sequence analysis of *pik3cd^E1017K^* knock-in events was direct Sanger sequencing of PCR products from primers L1_1017 and R2_1017 5′-gcaacatctccaaaagctgc-3′, using the PCR primers as sequencing primers.

ApoI digestion of PCR products used 0.5 µl (5 units) ApoI (NEB) directly added to 20 µl PCR reaction with 3 h incubation at 50°C.

### Tail-fin injury assays

The *TgBAC(mpx:gfp)i114* 3 dpf tail-fin injury inflammation time course assay followed Renshaw et al. (2006). Transection was through the posterior tip of the notochord with a scalpel blade. Injured embryos were kept alone in 24-well plates. Neutrophils posterior to the circulatory loop were counted at successive time points using a Leica M165FC fluorescent stereo microscope.

For the 5 dpf, Sudan Black *TgBAC(mpx:gfp)i114* assay, transection was of the posterior tip of the tail-fin with a scalpel blade. At 2 hpi, embryos were fixed for 1 h at room temperature in 4% paraformaldehyde (PFA) in PBS, rinsed three times with PBS, immersed for 20 min in Sudan Black B staining solution (Merck), 1 h in 70% ethanol with multiple changes, incrementally rehydrated into PBS with 0.1% Tween 20, 1 mM EDTA (PBSTwE), mounted in a µ-slide (four-well, glass-bottom) (ibidi) in 1% low-gelling temperature agarose in PBS and submerged in PBSTwE, then imaged in brightfield and a *z*-stack of fluorescent widefield with a Nikon Eclipse TE2000-U or Leica DMi8 inverted compound fluorescence microscope.

For the tail-fin regeneration assay, transection was through the posterior tip of the notochord with a scalpel blade at 3 dpf. At 5 dpf, embryos were mounted in 1% low-gelling temperature agarose in E3 medium, submerged in E3 medium and imaged with brightfield using a Nikon Eclipse TE2000-U or Leica DMi8 inverted compound microscope. The area of fin posterior to the notochord was manually traced and measured using Fiji.

For the 3 dpf *TgBAC(il1b:EGFP)sh445* assay, transection was of the posterior tip of the tail-fin with a scalpel blade. At 3 hpi, embryos were fixed for 90 min at room temperature in 4% PFA in PBS, rinsed with PBSTwE, washed into 0.1 M Tris-HCl pH 7.5, 0.15 M NaCl, 0.05% Tween 20, stained for 30 min in the dark without agitation in 1:50 Cyanine 5 Plus Amplification Reagent in Plus Amplification Dilutant (Perkin Elmer), washed into PBSTwE, mounted in a µ-slide (four-well, glass-bottom) (ibidi) in 1% low-gelling temperature agarose in PBS and submerged in PBSTwE. A Nikon CSU W1 microscope with 25× silicon immersion lens collected spinning disk confocal *z*-stack images. For each embryo, a single neutrophil in the fin posterior to the notochord was analysed from maximal-intensity projections of the *z*-stacks. The Cy5 channel was used to segment the neutrophil using NIS Elements (Nikon) automatic region selector. The GFP channel average fluorescence intensity was measured within the neutrophil's selected region using NIS Elements.

Time-lapse widefield video microscopy of *TgBAC(mpx:gfp)i114* neutrophil migration followed Isles et al. (2021). Embryos at 3 dpf were mounted in 1% low-gelling temperature agarose in E3 medium, submerged in E3 medium, and then a 30G syringe needle was used to cut a small notch on the ventral side of the tail-fin immediately before capturing a 6 h time-lapse series using a Nikon Eclipse TE2000-U inverted compound fluorescence microscope. Semi-automated tracking of fluorescent neutrophils used NIS Elements (version 4.3) with an additional NIS elements tracking module. Maximal-intensity projections were used to generate a binary layer that was smoothed, cleaned and separated to enable tracking. Neutrophils were only tracked if they reached the wound, and those neutrophils were tracked until they reached the wound.

### Whole-body neutrophil and macrophage counts

For light-sheet microscopy, *TgBAC(mpx:gfp)i114* or *Tg(mpeg1.1:NLS-clover)sh616* embryos were embedded in groups of three in 1% low-gelling temperature agarose in E3 medium in a mounting capillary, imaged with a ZEISS Light Sheet Z.1 and then individually collected for PCR genotyping. Each embryo was imaged with *z*-stacks through the head and trunk from each side and a *z*-stack through the tail. Fluorescent cells were manually counted from *z*-stacks using the Fiji point tool. Manual inspection ensured that cells were not double counted between the views.

For spinning disk microscopy of CAL-101-treated and control *TgBAC(mpx:gfp)i114* embryos, alternate groups of 24 treated or control embryos were mounted in the wells of a µ-slide (four-well, glass-bottom) (ibidi) in 1% low-gelling temperature agarose in E3 medium and submerged in E3 medium. A Nikon CSU W1 microscope with 10× lens was used to collect brightfield and a *z*-stack of spinning disk confocal images (350 µm range, 5 µm increments). Each embryo was imaged at three axial positions set automatically to create a merged tiled image. The set of embryos was imaged together as an automated multipoint series within a 2 h time window. Fluorescent cells were manually counted from a maximal-intensity projection of the fluorescent channel using the Fiji point tool. Every embryo was counted at the same brightness-contrast setting.

### WKM flow cytometry

WKM from adult *TgBAC(mpx:gfp)i114* zebrafish was dissected following Gerlach et al. (2011) and collected into 200 µl live sorting buffer [80% Leibovitz's L-15 medium (Thermo Fisher Scientific), 20% foetal bovine serum, 5 mM EDTA] on ice. Immediately before flow cytometry, WKM was macerated by pipetting, passed through a 40 µm cell strainer, rinsed through by addition of 400 µl PBS, and then 5 µl of 1 mM TO-PRO-3 (Thermo Fisher Scientific) stain was added. From each sample, 10^5^ cells were analysed with a BD FACSMelody cell sorter. Sorting was based on forward scatter and side scatter to identify leukocyte and precursor single cells following Traver et al. (2003), TO-PRO-3 fluorescence to exclude non-viable cells and GFP fluorescence to identify neutrophils. Analysis was performed using BD FACSChorus software (BD Biosciences).

### *S. aureus* infection

Infection assays followed Prajsnar et al. (2008). Embryos mounted in 3% methylcellulose (Merck) in E3 medium were injected, at 30 hpf, with 1500 colony-forming units of *S. aureus* (strain SH1000) in 1 nl of 2% polyvinylpyrrolidone (Merck) in PBS with Phenol Red (Merck), into the duct of cuvier. After soaking in E3 medium to release the embryos, undamaged embryos were transferred to individual wells of 96-well plates. They were inspected each morning and evening using a stereo microscope. Any without a heartbeat or with extensive necrosis were collected and stored at −20°C until genotyping.

### Statistics and image analysis

GraphPad Prism 9 was used for statistics and charts. Fiji was used for image analysis (Schindelin et al., 2012). Figures were prepared using Fiji and Adobe Photoshop.

## Supplementary Material

10.1242/dmm.049841_sup1 Supplementary information
