# Supplementary material for: Activated PI3K delta syndrome 1 mutations cause neutrophilia in zebrafish larvae
Source: Dis Model Mech. 2023 Mar 13;16(3):dmm049841. doi: 10.1242/dmm.049841 (PMC10655814; doi:10.1242/dmm.049841)
Supplement: Supplementary information [file dmm-16-049841-s1.pdf]

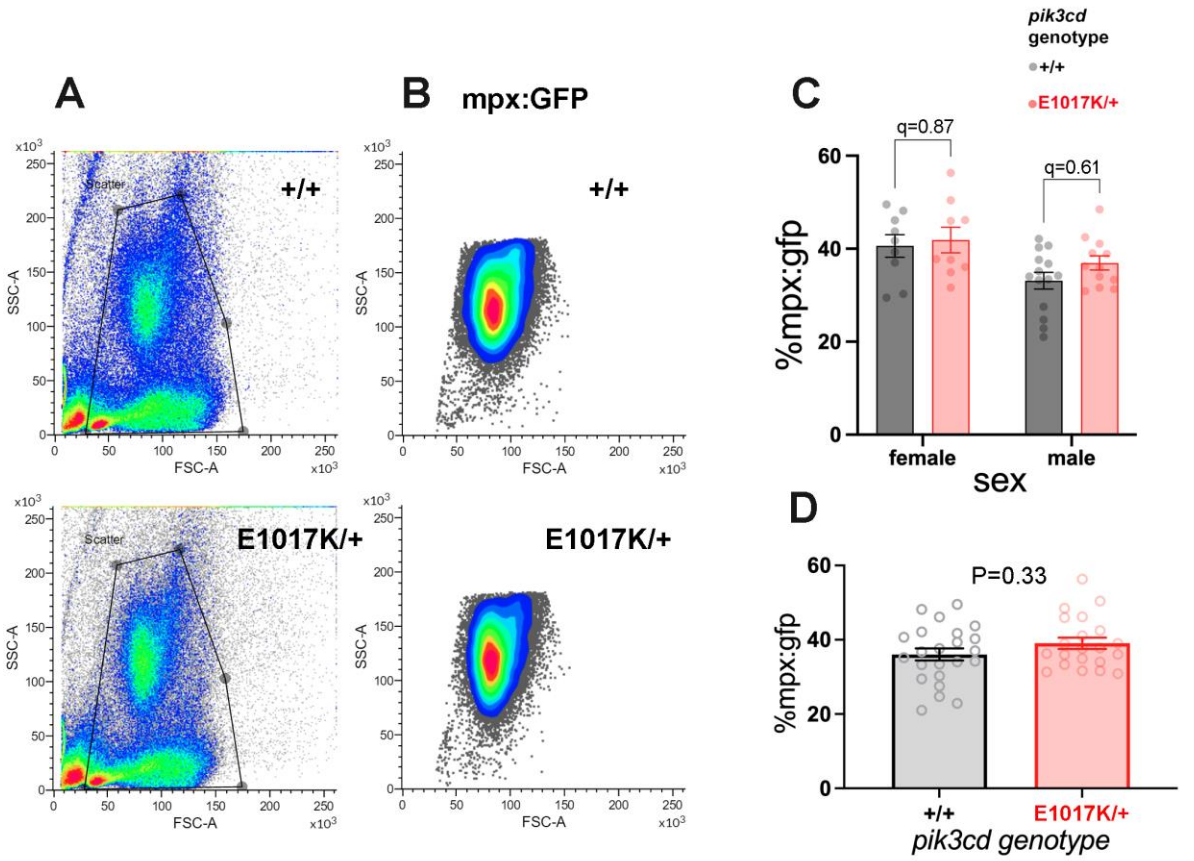

Fig. S1. Whole kidney marrow flow cytometry assessment of neutrophils in *pik3cd*$^{E1017K/+}$ adults. (A) Density plots of forward scatter and side scatter sorted whole kidney marrow from individual, eight month old, *TgBAC(mpx:gfp)i114*; *pik3cd*$^{E1017K/+}$ and *TgBAC(mpx:gfp)i114*; *pik3cd*$^{+/+}$ zebrafish. Gates are marked for the cell population analysed. (**B**) Density plots of GFP fluorescent viable cells from the cell population gated in (A). (**C**) Chart showing proportion of leukocyte and precursor cells as selected in (A) that are *TgBAC(mpx:gfp)i114* positive (as in (B)) from 21 *TgBAC(mpx:gfp)i114*; *pik3cd*$^{E1017K/+}$ and 23 *TgBAC(mpx:gfp)i114*; *pik3cd*$^{+/+}$ zebrafish, of the same age, raised together from the same clutches. Comparisons are shown for females and for males. Bars show the mean±SEM, Multiple Mann-Whitney tests. Single experiment. (**D**) The same data as in (C) but not segregated by sex. Bars show the mean±SEM, Mann-Whitney test.

Disease Models & Mechanisms • Supplementary information
